# Serum Urea-to-Albumin Ratio Is an Independent Predictor of Intra-Hospital Mortality in Neurosurgical Intensive Care Unit Patients with Spontaneous Intracerebral Hemorrhage

**DOI:** 10.3390/jcm12103538

**Published:** 2023-05-18

**Authors:** Michael Bender, Kristin Haferkorn, Shahin Tajmiri-Gondai, Marco Stein, Eberhard Uhl

**Affiliations:** Department of Neurosurgery, Justus-Liebig-University, 35392 Gießen, Germany; kristin.haferkorn@neuro.med.uni-giessen.de (K.H.); shahin.tajmiri-gondai@neuro.med.uni-giessen.de (S.T.-G.); marco.stein@neuro.med.uni-giessen.de (M.S.); eberhard.uhl@neuro.med.uni-giessen.de (E.U.)

**Keywords:** serum urea-to-albumin ratio, intracerebral hemorrhage, intra-hospital mortality, intensive care unit treatment, critical care, inflammation

## Abstract

The negative prognostic value of an increased serum urea-to-albumin ratio on intra-hospital mortality is frequently investigated in general critically ill patients and patients with septic shock, although not in neurosurgical patients with spontaneous intracerebral hemorrhages (ICH). The current study was conducted to investigate the impact of the serum urea-to-albumin ratio upon hospital admission on intra-hospital mortality in ICU-admitted neurosurgical patients with spontaneous ICH. Methods: This retrospective study analyzed 354 ICH patients, who were treated from 10/2008 to 12/2017 at our intensive care units (ICU). Blood samples were taken upon admission, and the patients’ demographic, medical, and radiological data were analyzed. A binary logistic regression analysis was performed for the identification of independent prognostic parameters for intra-hospital mortality. Results: Overall, the intra-hospital mortality rate was 31.4% (n = 111). In the binary logistic analysis, a higher serum urea-to-albumin ratio (OR = 1.9, CI = 1.23–3.04, *p* = 0.005) upon admission was identified as an independent predictor of intra-hospital mortality. Furthermore, a serum urea-to-albumin ratio cut-off level of >0.01 was associated with raised intra-hospital mortality (Youden’s index = 0.32, sensitivity = 0.57, specificity = 0.25). Conclusion: A serum urea-to-albumin ratio greater than 1.1 seems to be a prognostic marker to predict intra-hospital mortality in patients with ICH.

## 1. Introduction

Despite continuous advancements in intensive care units (ICU) and surgical treatment, spontaneous intracerebral hemorrhages (ICH) are still related to a mortality rate of up to 52% [1,2,3,4,5,6,7]. On account of this, the prognostication of intra-hospital mortality is an important part of daily business for ICU healthcare professionals. Currently, a larger volume and expansion of ICH, lower level of consciousness, lower Glasgow Coma Scale (GCS) score, advanced age, larger peri-hemorrhagic edema, and the presence of an intraventricular hemorrhage (IVH) and hydrocephalus are the most frequently used early predictors for mortality and poor outcome in ICH patients [8,9,10,11,12,13]. In addition to these well-known predictors, several studies have demonstrated the benefit of serum biomarkers, e.g., elevated levels of C-reactive protein (CRP), troponin I, cortisol, blood glucose, serum lactate, and white blood cell count, as well as CRP-to-albumin and fibrinogen-to-albumin ratios, to predict poor outcome and increased mortality in ICH patients [6,14,15,16,17,18,19,20].

Several studies indicated the positive association of a raised serum urea-to-albumin ratio with increased mortality in critically ill patients without chronic kidney diseases, septic shock or community-acquired pneumonia [21,22,23]. The serum urea level is affected by several different parameters, e.g., volume status, catabolism of endogenous proteins, liver function, trauma, nutritional status and/or glomerular filtration, and expresses the balance between production, metabolism and elimination [21,24,25]. Therefore, serum urea is not just a marker for single-organ dysfunction but a parameter for the severity of the underlying diseases and comorbidities. Furthermore, albumin is a relevant clinical parameter for the present nutritional status and liver synthesis function of the patient [11,14,26]. In addition, various studies have indicated that a lower albumin level is related to increased intra-hospital mortality after sepsis, septic shock, ICH and community-acquired bloodstream infections [11,26]. However, the impact of the serum urea-to-albumin ratio on predicting intra-hospital mortality in neurosurgical ICU-admitted patients with ICH is still unknown. The serum urea-to-albumin ratio could be a useful serum biomarker for the early identification of patients with an increased risk of intra-hospital mortality, and could therefore be helpful to improve further decisions on ICU treatment. Therefore, the current study was conducted to assess the impact of the serum urea-to-albumin ratio on intra-hospital mortality in neurosurgical ICU patients with spontaneous ICH.

## 2. Materials and Methods

### 2.1. Study Design and Population

**Figure 1:**
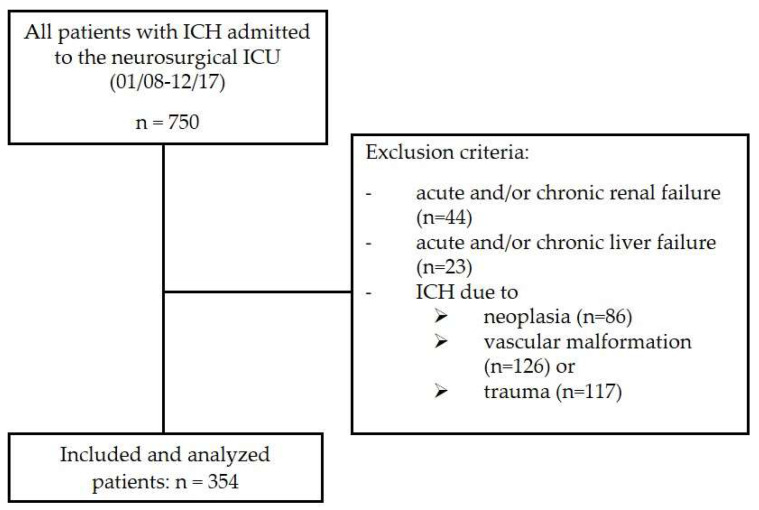
Flow chart for inclusion and exclusion criteria. ICH: intracerebral hemorrhage; ICU: intensive care unit treatment.

The present study analyzed all patients with spontaneous ICH who were treated at the neurosurgical ICU of the Neurosurgical Department of the University Hospital Giessen between October 2008 and December 2017 for at least 24 h (n = 750). The study protocol was approved by the Ethics Committee of Justus Liebig University, Giessen, Germany (No. 95/17). A computed tomography scan was performed on admission to confirm the diagnosis of ICH, and the serum urea level, as well as the albumin level, were determined from an initial blood sample taken from all patients. Exclusion criteria were defined as (1) the presence of chronic and/or acute renal failure (n = 44) and/or the evidence of chronic and/or acute liver failure (n = 23) due to direct damage or by secondary damage, e.g., cardiopulmonary decompensation or sepsis; (2) and age of <18 years; and (3) ICH due to neoplasia (n = 86), vascular malformation (n = 126) or trauma (n = 117), as presented in Figure 1 [27].

### 2.2. Data Collection

The baseline data, including sex, body mass index, age, and GCS, and duration of hospital stay, as well as serum biomarkers, comorbidities, premedication, radiological data upon admission and intra-hospital outcome, and mortality at discharge, were extracted from the patients’ electronic medical records and evaluated [28]. Comorbidities comprised the presence of cancer without cerebral manifestation, cardiac arrhythmia, diabetes mellitus, chronic arterial hypertension, coronary artery diseases, history of ischemic stroke or ICH, chronic obstructive pulmonary diseases, heart failure, and history of cardiac/cardiosurgical intervention. In addition, the long-term intake of antidiabetic, antiobstructive, antihypertensive, and antiplatelet agents as well as vitamin K antagonists and new oral anticoagulants were assessed as premedication.

### 2.3. Treatment Regime and Intensive Care Unit Treatment

After confirmation of the ICH diagnosis by a computed tomography scan, all patients were treated either immediately or after emergency surgery at the neurosurgical ICU for at least 24 h. The indication for medical or additional surgical treatment was made by a neurosurgery consultant in accordance with the clinical and radiological conditions of the ICH patients. The medical treatments comprised all modalities of conservative ICU treatments. Surgical treatments included the evacuation of ICH, insertion of an external ventricular drain (EVD), decompressive craniectomy or decompressive craniectomy with the evacuation of ICH. After admission to the ICU, all patients were monitored with a 3-lead electrocardiogram (B. Braun, Melsungen, Germany), an invasive blood pressure measurement catheter (Combitrans Monitoring Set arterial; B. Braun, Melsungen, Germany) and a pulse oximeter (Nellcor adult SpO_2_ sensor; Covidien LLC, Mansfield, MA, USA). A systolic blood pressure of 120 to 140 mmHg and an arterial oxygen partial pressure above 100 mmHg during the first 14 days were defined as cardiopulmonary targets. In addition, endotracheal intubation and mechanical ventilation were carried out in cases of respiratory insufficiency or a GCS score < 9 (Servo-I; Maquet, Rastatt, Germany). Therefore, propofol (200–500 mg/h) or midazolam (5–40 mg/h), in combination with sufentanil (35–100 µg/h), was used for continuous analgosedation.

### 2.4. Serum Biomarkers

Immediately after admission to the neurosurgical ICU, blood samples were routinely drawn in all patients. In general, blood glucose levels in mg/dL (ADVIA Chemistry XPT*^®^*, Siemens, Munich, Germany), the white blood cell count in giga/L (XE 5000 Hematology Analyzer, Sysmex, Norderstedt, Germany), serum lactate level in mmol/L (ADVIA Chemistry XPT*^®^*, Siemens, Germany), partial thromboplastin time in sec (Atellica*^®^* COAG 360 System, Siemens, Germany), hemoglobin level in g/dL (XE 5000 Hematology Analyzer, Sysmex, Germany), hematocrit level in % (XE 5000 Hematology Analyzer, Sysmex, Germany), creatinine in mg/dL (ADVIA Chemistry XPT*^®^*, Siemens, Germany), prothrombin time in % (Atellica*^®^* COAG 360 System, Siemens, Germany), cholinesterase in U/L (ADVIA Chemistry XPT*^®^*, Siemens, Germany), cortisol level in µg/dL (ADVIA Centaur XPT*^®^*, Siemens, Germany), C-reactive protein (CRP) in mg/L (ADVIA Chemistry XPT*^®^*, Siemens, Germany), serum urea in g/L (ADVIA Chemistry XPT*^®^*, Siemens, Germany), albumin level in g/L (ADVIA Chemistry XPT*^®^*, Siemens, Germany), and antithrombin III in %/NORM (Atellica*^®^* COAG 360 System, Siemens, Germany) were assessed as serum biomarkers in all patients. In addition, the serum urea-to-albumin ratio, as a mass concentration ratio, was calculated by the division of the initial serum urea level and the albumin level.

### 2.5. Radiological Data

The CT scan upon admission was assessed with regard to the localization of the ICH (infratentorial vs. supratentorial -lobar and deep-) and the volume of ICH, which was calculated by the formula A × B × C/2. Furthermore, the presence of hydrocephalus and IVH was confirmed by a Evans’ Index > 0.3 or Graeb score > 1 [29,30].

### 2.6. Intra-Hospital Mortality

The Modified Rankin Scale (mRS) at discharge was used for the analysis of the intra-hospital mortality and outcome [31].

### 2.7. Statistical Analysis

The total study population was stratified into non-survivors and survivors with respect to the intra-hospital outcome. Parameters with normal distributions are expressed as the mean ± standard deviation and the median and interquartile range (IQR) was used for non-normal distributed parameters. The univariate analysis was performed using the Student’s *t* test or Mann–Whitney U-Test and the Chi-square test to identify differences in binary variables between non-survivors and survivors. A *p*-value of <0.05 was defined as the level of significance. Furthermore, all parameters that reached the level of significance in the univariate analyses were further investigated in a binary logistic analysis with a forward stepwise method. A forward elimination method was performed for the selection of our variables. Therefore, the Statistical Package for the Social Sciences (SPSS) version 15.0 for Windows (Version 15.0; SPSS Inc., Chicago, IL, USA) was utilized for data analysis. Additionally, a cut-off level for the serum urea-to-albumin ratio level was calculated to predict increased intra-hospital mortality. The area under the curve and Youden’s index were calculated in a receiver operating curve analysis using R statistical software (Version 3.4.1, RCore Team 2017, Dormagen, Germany).

## 3. Results

### 3.1. Main Characteristics

The entire study population included 354 patients with a mean age of 68.6 ± 13.1 years (range: 18–93 years), out of whom 161 (45.5%) patients were women. The median GCS score upon admission was 8 (IQR: 3–12). A total of 211 (59.6%) patients required intubation and mechanical ventilation within the first 24 h. The most common comorbidities were arterial hypertension (58.8%) and cardiac arrhythmia (19.8%). Additionally, antihypertensive drugs (46.3%) and vitamin K antagonists (20.6%) were the most observed medications. Upon admission, the mean initial serum urea and albumin levels were 0.41 ± 0.22 g/L and 38.2 ± 5.4 g/L, respectively, resulting in a mean serum urea-to-albumin ratio of 0.01 ± 0.007. A total of 151 (42.7%) patients were treated conservatively, whereas 203 patients needed surgical treatment, of which the insertion of an external ventricular drain (34.4%) was the most frequently performed procedure. Mean ICH volume by computed tomography scan was 52.3 ± 42.2 cm^3^ (range: 1.0–219.6 cm^3^), and deep supratentorial (50.8%) was the most common ICH localization. Additionally, IVH was found in 248 (70.1%) patients, and 158 (44.6%) suffered from hydrocephalus. The main characteristics of all patients are summarized in Table 1.

### 3.2. Intra-Hospital Mortality

The median mRS at discharge of the entire study population was 5 (IQR:4–6) and 111 patients (31.4%) had died. In the univariate analysis, intra-hospital mortality was significantly associated with advanced age (*p* < 0.0001), lower initial GCS score (*p* < 0.0001), higher necessity of intubation within the first 24 h (*p* = 0.0002), lower body temperature upon admission (*p* < 0.0001), shorter length of hospital stay (*p* = 0.03), and heart failure (*p* = 0.02), as well as a lower rate of consumption of antihypertensive medication (*p* = 0.0009) and lower rate of pre-existing chronic arterial hypertension (*p* = 0.02). In addition, significantly lower levels of cholinesterase (*p* < 0.0001), prothrombin time (*p* = 0.01) and albumin (*p* < 0.0001) as well as higher levels of blood glucose (*p* = 0.007), partial thromboplastin time (*p* = 0.002), lactate (*p* = 0.01), C-reactive protein (*p* = 0.005), serum urea (*p* < 0.0001) and serum urea-to-albumin ratio (*p* < 0.0001) upon admission were found in the group of non-survivors. Moreover, infratentorial localization of ICH (*p* = 0.02), larger ICH volume (*p* < 0.0001) and higher evidence of IVH (*p* < 0.0001) and hydrocephalus (*p* = 0.003) were associated with intra-hospital mortality, as presented in Table 1.

**Figure 2:**
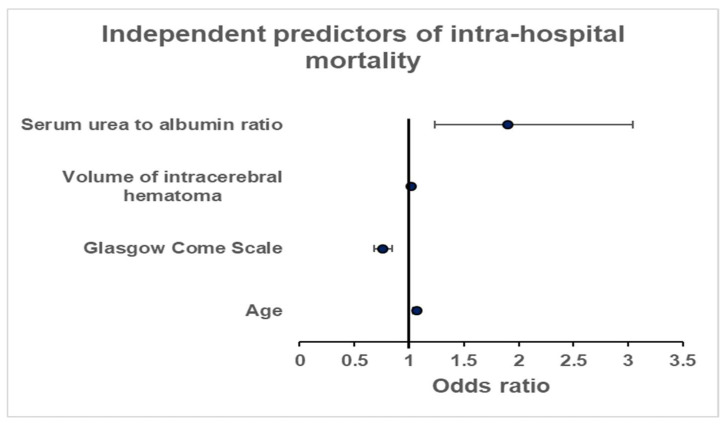
Independent predictors of intra-hospital mortality.

The binary logistic analysis identified lower GCS (odds ratio [OR] = 0.76, 95% confidence interval [CI] = 0.68–0.84, *p* < 0.0001), advanced age (OR = 1.07, CI:1.03–1.09, *p* < 0.0001), larger volume of intracerebral hematoma (OR = 1.02, CI = 1.01–1.02, *p* < 0.0001) and a higher serum urea-to-albumin ratio (OR = 1.9, CI = 1.23–3.04, *p* = 0.005) on admission as independent predictors of intra-hospital mortality in ICU-admitted neurosurgical patients with ICH (Figure 2).

Moreover, ROC analysis revealed a significant relationship between the initial serum urea-to-albumin ratio cut-off level of >0.01 and increased intra-hospital mortality (Youden’s index = 0.32, sensitivity = 0.57, specificity = 0.25).

## 4. Discussion

### 4.1. Summary of Findings

The current study investigated for the first time the impact of the serum urea-to-albumin ratio on intra-hospital mortality in neurosurgical patients with ICH treated on the ICU. In accordance with previous studies, lower GCS score, advanced age, and larger volume of ICH upon admission are independent predictors of intra-hospital mortality [2,3,4,5,7,8,9,10,11,15,18,19]. Additionally, the current study identified a raised serum urea-to-albumin ratio as a new independent predictor of intra-hospital mortality in this kind of patient. The impact of serum urea-to-albumin ratio has been investigated in critically ill patients with non-chronic kidney diseases, septic shock and community-acquired pneumonia [21,22,23]. This report shows, to the best of our knowledge for the first time, a significant relationship between intra-hospital mortality and a serum urea-to-albumin ratio > 0.01 upon admission in neurosurgical patients with ICH. This finding could be helpful to assist early decision making with respect to initiating or declining further ICU treatment.

### 4.2. Intra-Hospital Mortality

The intra-hospital mortality rate in the current study was 31.4%, which is consistent with those of previous studies [2,3,4,5,7,11]. In accordance with several earlier studies, lower initial GCS score, higher necessity of intubation within the first 24 h, advanced age, lower body temperature upon admission, a lower rate of consumption of antihypertensive medication and lower rate of pre-existing chronic arterial hypertension, heart failure, shorter length of hospital stay, infratentorial localization of ICH, larger volume of ICH volume, and a higher evidence of IVH and hydrocephalus, as well as lower levels of cholinesterase, albumin, prothrombin time and albumin, and higher levels of blood glucose, partial thromboplastin time, lactate, and C-reactive protein upon admission, were associated with intra-hospital mortality [1,2,3,6,7,8,9,10,11,12,13,14,15,18,19,32,33].

### 4.3. Serum Urea-to-Albumin Ratio

The serum urea-to-albumin ratio is an easy- and cheap-to-determine serum biomarker. The serum urea level is an elementary parameter of human metabolism, which is affected by a heterogenous number of parameters (e.g., volume status, catabolism of proteins, trauma, and nutritional status as well as liver function and/or glomerular filtration). In general, the total serum urea level is an expression of current metabolic status with respect to production, metabolism and elimination of serum urea in several organ systems, e.g., the liver, kidney, and gastrointestinal tract [21,24,25]. Therefore, serum urea could be applied as a surrogate parameter for the severity of the underlying diseases and comorbidities and not only as a parameter for a single-organ dysfunction. On this account, several studies reported the association of elevated serum urea levels and increased mortality in patients with hip fractures, small lung cell cancer, acute strokes, and patients with ischemic strokes which were treated with intravenous tissue plasminogen activators [34,35,36,37]. In contrast, negative acute-phase protein albumin is an important clinical indicator of current nutrition status and liver synthesis function of patients, and an appropriate biomarker for the prediction of inflammatory diseases and mortality after septic shock and ICH as well as community-acquired bloodstream infections [11,14,26]. However, the total level of both biomarkers, serum urea and albumin, is affected by various factors, e.g., hypovolemia, trauma, malnutrition, cancer, and catabolism as well as hepatic or renal insufficiency. Therefore, the serum urea-to-albumin ratio was implemented to determine the severity of the underlying diseases including trauma, cancer, malnutrition, hypovolemia, and systemic inflammation, as well as hepatic and/or renal insufficiency [21,22,23]. Several studies reported the positive association of an elevated serum urea-to-albumin ratio with increased mortality in critically ill patients with non-chronic kidney diseases, septic shock and community-acquired pneumonia; however, heterogenous cut-off levels have been described [21,22,23]. Pereira et al. identified a cut-off level of 47.25 for increased mortality in patients with septic shock [21]. In contrast, Gundpatil et al. found that a cut-off level of 23.44 predicts increased mortality in ICU-admitted patients with non-chronic kidney disease, while in the present study a serum urea-to-albumin ratio cut-off level of >0.01 was related to an increased intra-hospital mortality in ICU-admitted neurosurgical patients with ICH [22]. This heterogeneity may be explained by the differences in the study population and the underlying disease. Patients admitted to a medical or general surgical ICU due to infection (e.g., pneumonia or sepsis) or after surgery (e.g., oncological surgery) are more likely suffering from greater systemic inflammation expressed in a higher serum urea, and lower albumin, level and hence, a higher serum urea-to-albumin ratio than ICU-admitted neurosurgical ICH patients, in which loss of consciousness is the main indication for admission.

The present study suggests that the serum urea-to-albumin ratio could be a useful serum biomarker to predict intra-hospital mortality in neurosurgical ICU patients with ICH. Therefore, the serum urea-to-albumin ratio may be helpful in the early identification of patients with ICH and an increased risk of intra-hospital mortality as well as an additional parameter concerning decision making with respect to initiating or declining further ICU treatment.

### 4.4. Limitations and Strengths of the Study

The present study has several limitations, so the findings should be interpreted with caution. Firstly, it is a retrospective study, with its well-known problems. Furthermore, no repeated measurement of serum urea and albumin, and thus repeated serum urea-to-albumin ratio, was available. The logistic model was built using an unbalanced dataset where one characteristic (survivors) was more than twice as large as the other characteristic (non-survivors). This attribute can affect the predictive ability of our model, which was actually reflected in the low sensitivity and specificity values. Despite this limitation, the findings of the present study are interesting, especially considering that this is the first report about the predictive value of the serum urea-to-albumin ratio concerning intra-hospital mortality in neurosurgical ICH patients. Nevertheless, a prospective study with a larger study population should be carried out to confirm the results of the current study. After validation of the results of the current study, the serum urea-to-albumin ratio could be implemented in daily business to improve prognostication of intra-hospital mortality in neurosurgical ICU patients with ICH. Another interesting aspect, which should be investigated in further prospective studies, is to develop a model which includes several serum biomarkers, e.g., troponin I, procalcitonin, c-reactive protein to albumin ratio, cortisol, etc. as well as scores, e.g., the ICH-score or GCS or NIHSS score, to predict intra-hospital mortality in these patients. Finally, the results cannot be used for other types of ICH, as patients with malignancy, vascular malformations, or trauma were excluded.

Unlike the limitations, the strength of the current study comprises the large study population with demographic, clinical, radiological, and laboratory chemistry data. Additionally, this is the first study to investigate the impact of the serum urea-to-albumin ratios on intra-hospital mortality among neurosurgical ICU-admitted patients with ICH.

## 5. Conclusions

The serum urea-to-albumin ratio seems to be a new independent predictor of intra-hospital mortality in neurosurgical ICH patients. A serum urea-to-albumin ratio > 0.01 upon admission was significantly related to an increased intra-hospital mortality. This finding could be a helpful additional parameter for the decision to withdraw or initiate further ICU treatment in ICU-admitted neurosurgical patients with ICH.

## Figures and Tables

**Table 1:** Main characteristics of the study population (n = 354).

Parameter	Overall (n = 354)	Survivor (n = 243)	Non-Survivor (n = 111)	*p*-Value
Baseline data				
Age, years, mean (±SD) *	68.6 (13.1)	66.8 (13.3)	72.8 (11.8)	<0.0001
Women, n (%) *	161 (45.5)	119 (49)	42 (37.8)	0.05
Men, n (%) *	193 (54.5)	124 (51)	69 (62.2)	
Body-Mass-Index, kg/m^2^, median (IQR) *	26.1 (24.2–29.3)	26.6 (24.2–29.4)	25.7 (23.7–27.8)	0.16
Glasgow Coma Scale score, median (IQR) *	8 (3–12)	10 (6–13)	4 (3–7)	<0.0001
Intubated patients, n (%) **	211 (59.6)	129 (53.1)	82 (73.9)	0.0002
Body temperature, centigrade, median (IQR) *	36.3 (35.5–36.9)	36.4 (35.8–37)	35.9 (35.1–36.6)	<0.0001
Hospital stay, median (IQR) ****	16.5 (4.8–27)	22 (13–33)	3 (1–8)	0.03
Comorbidities				
Chronic arterial hypertension, n (%) *	208 (58.8)	153 (63)	55 (49.5)	0.02
Chronic obstructive pulmonary diseases, n (%) *	15 (4.2)	10 (4.1)	5 (4.5)	0.87
Cardiac arrhythmia, n (%) *	70 (19.8)	45 (18.5)	25 (22.5)	0.38
Coronary artery disease, n (%) *	44 (12.4)	26 (10.7)	18 (16.2)	0.14
Heart failure, n (%) *	20 (5.6)	9 (3.7)	11 (9.9)	0.02
Diabetes mellitus, n (%) *	56 (15.8)	40 (16.5)	16 (14.4)	0.62
History of ischemic stroke, n (%) *	50 (14.1)	38 (15.6)	12 (10.8)	0.23
History of ICH, n (%) *	17 (4.8)	9 (3.7)	8 (7.2)	0.15
History of cancer without cerebral manifestation, n (%) *	29 (8.2)	18 (7.4)	11 (9.9)	0.43
Premedication				
Antihypertensive drugs, n (%) *	164 (46.3)	127 (52.3)	37 (33.3)	0.0009
Antiobstructive drugs, n (%) *	5 (1.4)	2 (0.8)	3 (2.7)	0.16
Antidiabetic drugs, n (%) *	36 (10.2)	23 (9.5)	13 (11.7)	0.52
Antiplatelet agents, n (%) *	50 (14.1)	34 (14)	16 (14.4)	0.92
New oral anticoagulants, n (%) *	13 (3.7)	6 (2.5)	7 (6.3)	0.08
Vitamin K antagonist, n (%) *	73 (20.6)	50 (20.6)	23 (20.7)	0.98
Biomarkers				
White blood cells, giga/L, mean (± SD) *	10.9 (4.4)	10.6 (4)	11.7 (5.3)	0.03
Hemoglobin, g/dL, mean (±SD) *	13.1 (2)	13.2 (1.9)	12.9 (2.1)	0.33
Hematocrit, %, mean (±SD) *	38.5 (5.5)	38.6 (5.2)	38.2 (5.9)	0.5
Cholinesterase, U/L, mean (±SD) *	7809.4 (2282.3)	8115.9 (2226.2)	7138.5 (2269.5)	<0.0001
Blood glucose, mg/dL, mean (±SD) *	163.8 (59.3)	158.1 (55.6)	176.2 (65.2)	0.007
Serum lactate, mmol/L, mean (±SD) *	1.7 (1.5)	1.6 (1.3)	2 (1.7)	0.01
Cortisol, µg/dL, mean (±SD) *	27.7 (19.4)	27.2 (19.4)	29.2 (19.3)	0.42
C-reactive protein, mg/L, mean (±SD) *	22 (39.3)	30.6 (46.2)	37.1 (17.3)	0.005
Creatinine, mg/dL, mean (±SD) *	0.9 (0.6)	0.9 (0.5)	1 (0.8)	0.61
Serum urea, g/L, mean (±SD) *	0.41 (0.22)	0.37 (0.17)	0.50 (0.27)	<0.0001
Albumin, g/L, mean (±SD) *	38.2 (5.4)	39 (4.8)	36.5 (6.3)	<0.0001
Serum urea-to-albumin ratio, mean (±SD) *	0.01 (0.007)	0.01 (0.005)	0.014 (0.009)	<0.0001
Prothrombin time, %, mean (±SD) *	83.7 (26.2)	86.1 (25)	78.4 (28.3)	0.01
Partial thromboplastin time, seconds, mean (±SD) *	32.5 (11.4)	31.3 (8.3)	35.3 (16.1)	0.002
Antithrombin III, %/NORM, mean (±SD) *	88.7 (16)	89.9 (14.4)	86.6 (18.3)	0.16
Treatment				
Medical treatment, n (%) ***	151 (42.7)	98 (40.3)	53 (47.7)	0.19
Surgical Treatment, n (%) ***	203 (57.3)	145 (59.7)	58 (52.3)	
Insertion EVD, n (%) ***	77 (37.9)	53 (36.6)	24 (41.4)	0.52
Evacuation ICH, n (%) ***	62 (30.5)	46 (31.7)	16 (27.6)	0.56
Decompressive craniectomy, n (%) ***	18 (8.9)	12 (8.3)	6 (10.3)	0.64
Decompressive craniectomy and Evacuation ICH, n (%) ***	46 (22.7)	34 (23.4)	12 (20.7)	0.67
Radiological data				
Localization				
Supratentorial, lobar, n (%) *	122 (34.5)	78 (32.1)	44 (39.6)	0.17
Supratentorial, deep, n (%) *	180 (50.8)	122 (50.2)	58 (52.3)	0.72
Infratentorial, n (%) *	52 (14.7)	43 (17.7)	9 (8.1)	0.02
ICH volume, cm^3^, mean (±SD) *	52.3 (42.2)	42.9 (34.9)	72.8 (49.1)	<0.0001
IVH, n (%) *	248 (70.1)	156 (64.2)	92 (82.9)	<0.0001
Hydrocephalus, n (%) *	158 (44.6)	94 (38.7)	64 (57.7)	0.003
mRS score, median (IQR) ****	5 (4–6)	4 (4–5)	-	

SD: standard deviation, IQR: interquartile range, ICH: intracerebral hemorrhage, EVD: external ventricular drain, IVH: intraventricular hemorrhage, mRS: modified Rankin Scale. The *p*-values refer to the comparison of the survivor with non-survivor groups. The univariate analysis was performed using Student’s *t* test or Mann–Whitney U-Test and the Chi-square test to identify differences in binary variables between survivors and non-survivors. * upon admission, ** within the first 24 h, *** during inpatient treatment, **** at discharge.

## Data Availability

The data presented in this study are available on request from the corresponding author.

## References

[1] Hays A., Diringer M.N. (2006). Elevated troponin levels are associated with higher mortality following intracerebral hemorrhage. Neurology.

[2] Stein M., Luecke M., Preuss M., Boeker D.K., Joedicke A., Oertel M.F. (2010). Spontaneous intracerebral hemorrhage with ventricular extension and the grading of obstructive hydrocephalus: The prediction of outcome of a special life-threatening entity. Neurosurgery.

[3] Hjalmarsson C., Bergfeldt L., Bokemark L., Manhem K., Andersson B. (2013). Electrocardiographic abnormalities and elevated cTNT at admission for intracerebral hemorrhage: Predictors for survival?. Ann. Noninvasive Electrocardiol..

[4] Ahn C.S., Lee S.K., Kim H.S., Kong M.H., Song K.Y., Kang D.S. (2004). Surgical outcome of spontaneous intracerebral hemorrhage in less than stuporous mental status. J. Korean Neurosurg. Soc..

[5] Martí-Fàbregas J., Belvís R., Guardia E., Cocho D., Muñoz J., Marruecos L., Martí-Vilalta J.L. (2003). Prognostic value of Pulsatility index in acute intra-cerebral hemorrhage. Neurology.

[6] Garrett M.C., Komotar R.J., Starke R.M., Doshi D., Otten M.L., Connolly E.S. (2010). Elevated troponin levels are predictive of mortality in surgical intracerebral hemorrhage patients. Neurocrit. Care.

[7] Caplan L.R. (1992). Intracerebral haemorrhage. Lancet.

[8] Tuhrim S., Horowitz D.R., Sacher M., Godbold J.H. (1999). Volume of ventricular blood is an important determinant of outcome in supratentorial intracerebral hemorrhage. Crit. Care Med..

[9] Juvela S. (1995). Risk factors for impaired outcome after spontaneous intracerebral hemorrhage. Arch. Neurol..

[10] Davis S.M., Broderick J., Hennerici M., Brun N.C., Diringer M.N., Mayer S.A., Begtrup K., Steiner T., Recombinant Activated Factor VII Intracerebral Hemorrhage Trial Investigators (2006). Hematoma growth is a determinant of mortality and poor outcome after intracerebral hemorrhage. Neurology.

[11] Bender M., Haferkorn K., Friedrich M., Uhl E., Stein M. (2020). Impact of Early C-Reactive Protein/Albumin Ratio on Intra-Hospital Mortality among Patients with Spontaneous Intracerebral Hemorrhage. J. Clin. Med..

[12] Foerch C., Curdt I., Yan B., Dvorak F., Hermans M., Berkefeld J., Raabe A., Neumann-Haefelin T., Steinmetz H., Sitzer M. (2006). Serum glial fibrillary acidic protein as a biomarker for intracerebral haemorrhage in patients with acute stroke. J. Neurol. Neurosurg. Psychiatry.

[13] Gerner S.T., Auerbeck K., Sprügel M.I., Sembill J.A., Madžar D., Gölitz P., Hoelter P., Kuramatsu J.B., Schwab S., Huttner H.B. (2018). Peak Troponin I Levels Are Associated with Functional Outcome in Intracerebral Hemorrhage. Cerebrovasc. Dis..

[14] Bender M., Haferkorn K., Tajmiri-Gondai S., Uhl E., Stein M. (2022). Fibrinogen to Albumin Ratio as Early Serum Biomarker for Prediction of Intra-Hospital Mortality in Neurosurgical Intensive Care Unit Patients with Spontaneous Intracerebral Hemorrhage. J. Clin. Med..

[15] Yang X., Ren W., Zu H., Dong Q. (2014). Evaluate the serum cortisol in patients with intracerebral hemorrhage. Clin. Neurol. Neurosurg..

[16] Fonseca S., Costa F., Seabra M., Dias R., Soares A., Dias C., Azevedo E., Castro P. (2021). Systemic inflammation status at admission affects the outcome of intracerebral hemorrhage by increasing perihematomal edema but not the hematoma growth. Acta Neurol. Belg..

[17] Bender M., Haferkorn K., Nagl J., Uhl E., Stein M. (2022). Serum Lactate as Serum Biomarker for Cardiopulmonary Parameters within the First 24 Hours after a Spontaneous Intracerebral Hemorrhage. Diagnostics.

[18] Diedler J., Sykora M., Hahn P., Rupp A., Rocco A., Herweh C., Steiner T. (2009). C-reactive-protein levels associated with infection predict short- and long-term outcome after supratentorial intracerebral hemorrhage. Cerebrovasc. Dis..

[19] Agnihotri S., Czap A., Staff I., Fortunato G., McCullough L.D. (2011). Peripheral leukocyte counts and outcomes after intracerebral hemorrhage. J. Neuroinflamm..

[20] Tibaut M., Caprnda M., Kubatka P., Sinkovič A., Valentova V., Filipova S., Gazdikova K., Gaspar L., Mozos I., Egom E.E. (2019). Markers of Atherosclerosis: Part 2-Genetic and Imaging Markers. Heart Lung Circ..

[21] Pereira A.G., Costa N.A., Gut A.L., Azevedo P.S., Tanni S.E., Mamede Zornoff L.A., Rupp de Paiva S.A., Polegato B.F., Minicucci M.F. (2021). Urea to albumin ratio is a predictor of mortality in patients with septic shock. Clin. Nutr. ESPEN.

[22] Gundpatil D.B., Somani B.L., Saha T.K., Banerjee M. (2014). Serum urea: Albumin ratio as a prognostic marker in critical patients with non-chronic kidney disease. Indian J. Clin. Biochem..

[23] Ugajin M., Yamaki K., Iwamura N., Yagi T., Asano T. (2012). Blood urea nitrogen to serum albumin ratio independently predicts mortality and severity of community-acquired pneumonia. Int. J. Gen. Med..

[24] Beier K., Eppanapally S., Bazick H.S., Chang D., Mahadevappa K., Gibbons F.K., Christopher K.B. (2011). Elevation of blood urea nitrogen is predictive of long-term mortality in critically ill patients independent of “normal” creatinine. Crit. Care Med..

[25] Faisst M., Wellner U.F., Utzolino S., Hopt U.T., Keck T. (2010). Elevated blood urea nitrogen is an independent risk factor of prolonged intensive care unit stay due to acute necrotizing pancreatitis. J. Crit. Care.

[26] Artero A., Zaragoza R., Camarena J.J., Sancho S., González R., Nogueira J.M. (2010). Prognostic factors of mortality in patients with community-acquired bloodstream infection with severe sepsis and septic shock. J. Crit. Care.

[27] Levey A.S., Coresh J., Balk E., Kausz A.T., Levin A., Steffes M.W., Hogg R.J., Perrone R.D., Lau J., Eknoyan G. (2003). National Kidney Foundation practice guidelines for chronic kidney disease: Evaluation, classification, and stratification. Ann. Intern. Med..

[28] Teasdale G., Jennett B. (1974). Assessment of coma and impaired consciousness. A practical scale. Lancet.

[29] Evans W.A. (1942). An encephalographic ratio for estimating ventricular enlargement and cerebral atrophy. Arch. Neurol. Psychiatry.

[30] Graeb D.A., Robertson W.D., Lapointe J.S., Nugent R.A., Harrison P.B. (1982). Computed tomographic diagnosis of intraventricular hemorrhage. Etiology and prognosis. Radiology.

[31] van Swieten J.C., Koudstaal P.J., Visser M.C., Schouten H.J., van Gijn J. (1988). Interobserver agreement for the assessment of handicap in stroke patients. Stroke.

[32] Castellanos M., Leira R., Tejada J., Gil-Peralta A., Dávalos A., Castillo J. (2005). Stroke Project, Cerebrovascular Diseases Group of the Spanish Neurological Society. Predictors of good outcome in medium to large spontaneous supratentorial intracerebral haemorrhages. J. Neurol. Neurosurg. Psychiatry.

[33] Hemphill J.C., Greenberg S.M., Anderson C.S., Becker K., Bendok B.R., Cushman M., Fung G.L., Goldstein J.N., Macdonald R.L., Mitchell P.H. (2015). Guidelines for the Management of Spontaneous Intracerebral Hemorrhage: A Guideline for Healthcare Professionals from the American Heart Association/American Stroke Association. Stroke.

[34] Li C.Y., Zhang X.C., Zheng X., Rui M., Yao S.C., Song Z.J., Zhao F.C., Guo K.J. (2017). Effect of admission blood urea and creatinine levels on mortality in elderly patients with hip fracture. Zhongguo Gu Shang.

[35] Winter M.C., Potter V.A., Woll P.J. (2008). Raised serum urea predicts for early death in small cell lung cancer. Clin. Oncol. (R. Coll. Radiol.).

[36] Zhang Y., Churilov L., Meretoja A., Teo S., Davis S.M., Yan B. (2013). Elevated urea level is associated with poor clinical outcome and increased mortality post intravenous tissue plasminogen activator in stroke patients. J. Neurol. Sci..

[37] Idris I., Hill R., Sharma J.C. (2010). Effects of admission serum urea, glomerular filtration rate, proteinuria and diabetes status on 3-month mortality after acute stroke. Diabetes Vasc. Dis. Res..

